# While neither universally applicable nor practical operationally, the biological species concept continues to offer a compelling framework for studying species and speciation

**DOI:** 10.1093/nsr/nwaa108

**Published:** 2020-06-09

**Authors:** Lexuan Gao, Loren H Rieseberg

**Affiliations:** CAS Center for Excellence in Molecular Plant Sciences, Institute of Plant Physiology and Ecology, Chinese Academy of Sciences, China; Department of Botany and Biodiversity Research Centre, University of British Columbia, Canada; Department of Botany and Biodiversity Research Centre, University of British Columbia, Canada

Few topics in evolutionary biology have received as much scrutiny and debate as the nature of species and species concepts. This is partly because species are central to many fields of biology (but especially evolution, ecology, systematics, and conservation biology), and there does not appear to be a single concept that can satisfy the needs of these different disciplines [1]. A related issue is that the nature of species varies widely across the domains of life due to differences in mode of reproduction (sexual versus asexual), life cycle, generation time, mating system, vagility, genome architecture, geographic range size, and a host of other features [2]. Species concepts that can accommodate this natural diversity [3,4] offer little guidance regarding how to delimit species, study their origins and evolution, or predict their evolutionary fate. As a consequence, users of species concepts typically choose the concept that suits their specific requirements, often with the recognition that their favored concept has known flaws, lacks universality or both.

The biological species concept (BSC) is a good example of this [5]. Its flaws are well known [2,6–8]; it flunks the universality test and is impractical operationally. However, the BSC offers a compelling framework for studying speciation, so evolutionary geneticists continue to use it. But should we? This is the issue Wang *et al.* [9] address in their critique. They summarize what is known about the geography and genetics of speciation and come to the surprising conclusion that we lack sufficient knowledge to reject the BSC.

We come to a different conclusion. In our view, there is ample information about the nature of species to reject a strict version of the BSC. Moreover, the BSC’s liabilities when studying groups that are primarily asexual or that have a selfing mating system are well known [2]. Despite these limitations, we argue that the BSC continues to be a useful concept for studies of speciation in sexual and predominately outcrossing lineages. Below we highlight several points of agreement with Wang *et al.* [9], as well as several areas of disagreement. We conclude by describing our rationale for continuing to use the BSC, despite its flaws.

## POINTS OF AGREEMENT

We are in agreement with many of the points the authors have made about speciation in previous papers and that are summarized in their critique. We agree that species’ genomes are less tightly co-adapted than suggested by Mayr [5], and as a consequence, can be porous to gene flow when in sympatry or parapatry. We note that numerous hybrid zone studies over the past several decades have offered robust support for both conclusions [10–12]. Likewise, commonly reported patterns of heterogeneous genomic divergence are consistent with both differential introgression following secondary contact and divergence with gene flow [9,13]. Though it is important to keep in mind that recent selective sweeps in the absence of gene flow between populations can produce similar patterns [14].

The authors also offer useful insights here and elsewhere about the geography of speciation. They argue that gene flow during the early stages of speciation is compatible with Mayr's classic allopatric model of speciation. Using a case study of mangroves from the Indo-Malayan coast they further observe that allopatry is often intermittent, in this example due to the repeated openings and closures of the Strait of Malacca [15]. Lastly, they make the point that diversification rates in mangroves and many other taxa are poorly correlated with geographic features that can cause isolation, implying that geographical isolation is unlikely to be necessary for speciation in these groups. We agree with these assertions and note that the role of intermittent allopatry in speciation has been explored previously in the context of Quaternary ice ages, in which genomes persisting in refugia are thought to have undergone repeated cycles of allopatry and secondary contact, protected in part by hybrid zones [16]. More generally, the importance of geographic isolation appears to correlate both with vagility and with the strength of habitat associations. For example, sister species of plants, but not birds, are frequent on isolated oceanic islands [17,18]. The most straightforward explanation for this difference is that geographical isolation is necessary for speciation in birds, but not for many lineages of plants (see also ref. [19]).

## POINTS OF DISAGREEMENT

While this critique is a stimulating read, we disagree with the use of the BSC to define both a process and a concept of species. This confounds the products of speciation (i.e. species) with the mechanisms of speciation, and potentially constrains our views about how speciation can occur. Also, bear in mind that biological barriers to gene flow (reproductive barriers) typically are the result of speciation, not its cause [20]. This is most obviously true for species that have diverged exclusively in allopatry. Thus, reproductive barriers are best interpreted as indicators of progress towards speciation [1]. The BSC is (or at least should be) agnostic about the geographic context of speciation or the evolutionary forces responsible for the evolution of reproductive barriers. That is, implementation of the BSC does not (and should not) depend on whether a given species diverged in sympatry or allopatry, or whether reproductive barriers arose mainly as a consequence of genetic drift, divergent natural selection, or by whole genome duplication.

The authors argue that evidence of gene flow between good species with strong reproductive isolation (RI) would be sufficient to reject the BSC, but that such evidence does not yet exist. We were surprised by the latter claim, especially given that an increasing number of studies in plants and animals have not only measured the strength of RI in nature, but also have quantified levels of interspecific gene flow, in some cases across extremely strong reproductive barriers [21–24]. An example comes from a pair of widespread sunflower species, *Helianthus annuus* and *H. petiolaris*, which have overlapping geographic distributions across much of Central and Western North America. The two species diverged circa 1.8 mya and are strongly isolated reproductively [23]; total isolation—calculated by compounding the contributions of eight individual barriers—was >0.99999 in both directions. Thus, these are very good species! The interspecific migration rate (m) estimated from population genetic data is very small (<10^−7^), as expected given the strength of RI. However, because these species have very large effective population sizes (Ne > 10^6^), the predicted number of migrants per generation is high enough (N_e_m = 0.34–0.76) to produce mosaic genomes.

A final point of disagreement concerns the sensitivity of current genomic methods for detecting small introgressions. While we agree that the power for identifying individual introgressions is reduced when they are small, if there are many small introgressions across the genome, then the signature of interspecific gene flow is easily and robustly detected by various whole genome tests such as Patterson's D statistic [25] or from programs for identifying population structure such as Structure [26] or Admixture [27].

## CONCLUSION

If the BSC is faulty, then why do evolutionary geneticists continue to use it? Likewise, how can a focus on the evolution of reproductive barriers be justified? As we noted in the introduction, the main reason that evolutionary geneticists continue to use the BSC is that it offers a powerful framework for studying speciation. We previously documented a strong, albeit imperfect, correlation between the strength of RI and species delimitation by taxonomists [28], which offers empirical support for such a research program. On the other hand, we recognize that multiple evolutionary forces contribute to species identity and cohesion, including gene flow/RI, common descent, stabilizing and parallel selection, and genetic constraints [2]. Some of these likely correlate as strongly with taxonomic species as does RI, perhaps suggesting that students of speciation should expand the focus of their research beyond RI. However, as we have argued elsewhere [29], gene flow and RI are population and species-level phenomena, the levels of divergence relevant to speciation studies. In contrast, selection acts most strongly on genes or individuals, whereas common descent and genetic constraints contribute to cohesion across all taxonomic levels. In our view, this justifies a focus on gene flow/RI, while recognizing that studies which seek to comprehensively identify and order the evolutionary forces contributing to species cohesion would add significantly to our understanding of both species and speciation.

***Conflict of interest statement***. None declared.
